# Effect of Targeted Aspirin Administration Based on First Trimester Combined Screening for Preeclampsia on Preterm Birth: An Interrupted Time Series Analysis

**DOI:** 10.1111/1471-0528.70100

**Published:** 2025-11-24

**Authors:** Monica Minopoli, Alicia Martínez‐Varea, Claire Pegorie, Pilar Palmrich, Ana Pinas, Basky Thilaganathan

**Affiliations:** ^1^ Fetal Medicine Unit St George's University Hospitals NHS Foundation Trust London UK; ^2^ Department of Obstetrics and Gynaecology La Fe University and Polytechnic Hospital Valencia Spain; ^3^ Department of Obstetrics and Gynaecology Medical University of Vienna Vienna Austria; ^4^ Vascular Biology Research Centre, Molecular and Clinical Sciences Research Institute St George's University of London London UK

**Keywords:** first trimester, interrupted time series analysis, preeclampsia, preterm birth, screening

## Abstract

**Objectives:**

To assess the effect of an aspirin intervention program on preterm birth (PTB) rates in women identified as high‐risk for preterm preeclampsia in the first‐trimester screening.

**Design:**

This is a retrospective study evaluating the impact of the intervention program on preterm birth using interrupted time series analysis (ITSA) with an ARIMA model that accounts for seasonality and autocorrelation.

**Setting:**

St George's University Hospital, data collected on pregnancies between 2016 and 2022.

**Population:**

Pregnant women followed from the first trimester of pregnancy.

**Methods:**

An ITSA was conducted on the studied population to assess whether the introduction of an aspirin intervention program, based on first‐trimester screening, influenced preterm birth rates.

**Main Outcome Measures:**

Primary outcomes included overall PTB rates, while secondary outcomes included iatrogenic preterm birth (iPTB) and spontaneous preterm birth (sPTB).

**Results:**

Among 31 534 births, there were 1435 PTBs: 734 spontaneous and 701 iatrogenic. ITSA analysis revealed no significant impact of the screening program on PTB rates. There was a nonsignificant decrease in total PTB and iPTB over time, with similar trends observed for early PTB (< 34 weeks).

**Conclusions:**

First‐trimester screening for preterm preeclampsia identifies women at higher risk for both preterm preeclampsia and PTB. While aspirin prophylaxis and serial assessments improve some perinatal outcomes, they do not significantly reduce spontaneous or iatrogenic PTB rates. Additional interventions will be needed to achieve a substantial reduction in PTB rates in a high‐risk population.

## 1 Introduction

The first‐trimester combined screening test for preeclampsia, developed by the Fetal Medicine Foundation (FMF), has been implemented in various settings and has demonstrated superiority over the ACOG and NICE guidelines in correctly identifying patients at risk of preterm preeclampsia [1, 2, 3]. It has also proven effective in identifying other pregnancy complications such as stillbirth, small for gestational age (SGA) birth, and preterm birth (PTB) [4, 5, 6, 7]. Previous studies have shown that a screening program based on the first trimester combined risk test for preeclampsia, with interventions including first‐trimester aspirin administration, serial growth scans, and induction of labour at 40 weeks, can reduce the rate of preterm preeclampsia by up to 70% [8], improve detection of SGA fetuses [6] and prevent stillbirth, especially in women of minority ethnic origins [4]. Epidemiological evidence shows that aspirin prophylaxis may also reduce spontaneous PTB by approximately 10% [9]. Given these improvements in pregnancy health, there is an expectation that both spontaneous and iatrogenic PTB rates may potentially be reduced by systematic first‐trimester combined risk test for preeclampsia with targeted intervention in high‐risk women. The aim of this study is to analyse the impact of the introduction of the first trimester combined risk test for preeclampsia program on both iatrogenic and spontaneous PTB rates using interrupted time series analysis (ITSA).

## 2 Methods

This retrospective study was conducted at St George's University Hospital NHS Foundation Trust, on pregnancies followed between April 2016 and November 2022. In March 2018, a screening and intervention program aimed at reducing preterm preeclampsia was introduced. This program included daily administration of 150 mg of aspirin until 36 weeks, an additional ultrasound at 28 and 36 weeks, and induction of labour at 40 weeks for women identified as high‐risk. Risk assessment is performed using the Fetal Medicine Foundation algorithm, which incorporates maternal factors, mean arterial pressure (MAP), uterine artery pulsatility index (UtA‐PI), and pregnancy‐associated plasma protein‐A (PAPP‐A). Women with a risk greater than 1 in 50 are classified as high‐risk and offered this preventive strategy to reduce adverse pregnancy outcomes. Women whose first hospital encounter occurred after 16 weeks of gestation were excluded as they were not managed according to this screening program.

### 2.1 Study Variables and Outcome Measures

The primary outcome of the study was to assess the impact of the first‐trimester screening program based on prophylactic aspirin administration on preterm delivery. The secondary outcomes were iatrogenic preterm birth (iPTB), defined as preterm birth resulting from indicated induction of labour or Caesarean section due to fetal and/or maternal complications, and spontaneous preterm birth (sPTB) due to spontaneous preterm labour or preterm premature rupture of membranes (PPROM). Deliveries occurring before 37 weeks of gestation were classified as preterm, and those occurring before 34 weeks were classified as early preterm. Time‐series data, presented in quarters (Q1–Q4), were collected. Maternal demographic characteristics, past medical history, and prenatal data were obtained from the hospital's ultrasound database (ViewPoint version 5.6.26.148, ViewPoint Bildverarbeitung GmbH, Wessling, Germany). Delivery outcomes, including gestational age, birth weight, and mode of delivery, were obtained from the maternity birth registry (EuroKing, Wellbeing Software, Mansfield, UK). The local ethics committee advised that formal ethical approval was not required for this retrospective study.

### 2.2 Statistical Analysis

Descriptive data were represented by median and interquartile range for continuous variables and by number and percentage for categorical variables. The Wilcoxon rank‐sum test was used to compare continuous variables that were not normally distributed, while an independent *t*‐test was applied for normally distributed continuous variables. The Chi‐square test was used to analyse differences in categorical or binary variables between groups. An ITSA was performed to assess the impact of the first trimester combined screening program on the PTB rate [10]. The time series consists of quarterly reported rates of totals, spontaneous and iatrogenic preterm and early PTBs that occurred between April 2016 and November 2022. The screening program was implemented in St George's Hospital in March 2018. However, the intervention point was established in July 2018 to allow for the time lag between the start of the screening (first trimester) and the actual outcome of PTB from 24 weeks' gestation. The ITSA was conducted using an Autoregressive Integrated Moving Average (ARIMA) that accounts for seasonality and autocorrelation. This model assesses how the aspirin‐based first‐trimester combined screening program for preterm preeclampsia affected the PTB rate by analysing trends before and after the intervention using segmented regression. Specifically, it examines both the immediate effect of the intervention on the outcome and the long‐term effect by measuring how the trend changes over time. The ARIMA (1,0,0) model was assessed to be the best fit for the time series data. Statistical analysis was performed using Statistical Package for Social Sciences (SPSS) version 28.0.1.0 (IBM Inc.) and Stata (18.5 SE standard edition).

## 3 Results

**FIGURE 1:**
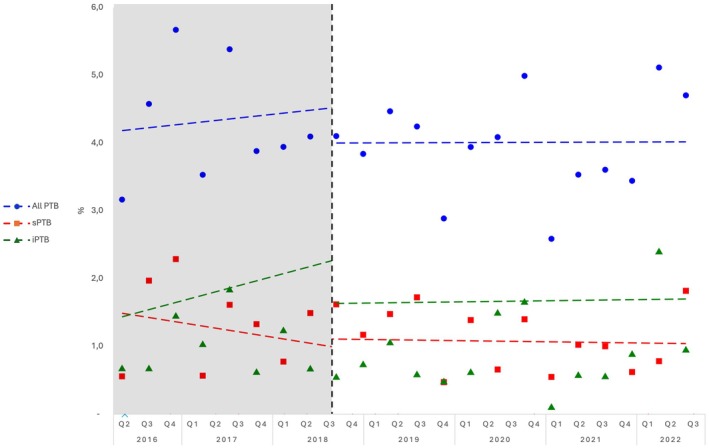
Time series of PTB, iPTB, and sPTB rates presented in quarters of years (Q1–Q4) before and after the introduction of the first trimester combined screening program for preterm preeclampsia. The grey‐shaded area represents the pre‐intervention period (April 2016–July 2018). The dashed vertical line marks the introduction of the screening program. The dotted trend lines represent the trajectory of PTB, sPTB, and iPTB rates before and after the intervention. The change in intercept at the time of intervention reflects the immediate effect of the program, while the post‐intervention trend indicates any long‐term changes in PTB rates, as assessed through Interrupted Time Series Analysis (ITSA). iPTB, iatrogenic preterm birth; PTB, preterm birth; sPTB, spontaneous preterm birth.

There was a total of 31 534 births, with 1435 (4.5%) PTBs (734 spontaneous and 701 iatrogenic) and 488 (1.5%) early PTBs. Demographic characteristics of the PTB population before and after the introduction of the first trimester combined risk test for preeclampsia program are presented in Table 1. The rates and trends of PTB before and after the introduction of the screening and intervention program are presented in Figure 1 and Figure S1. The ITSA showed that the introduction of aspirin prophylaxis based on the first trimester combined screening for preterm preeclampsia contributed to a nonsignificant immediate decrease of 0.21% and a nonsignificant reduction in the trend of PTB rates (Table 2, Figure 1). Similarly, the intervention resulted in a nonsignificant long‐term reduction in iPTB and an increase in sPTB (Table 2, Figure 1). Similar nonsignificant findings were seen for early PTB < 34 weeks (Table S1, Figure S1).

**TABLE 1:** Characteristics of the preterm birth population (*n* = 1435) before and after the introduction of the first‐trimester screening program for preterm preeclampsia.

	Pre‐intervention (*n* = 524)	Post‐intervention (*n* = 911)	*p*
Age at booking	33 (29–36)	32 (29–36)	0.898
Weight at booking (kg)	65.0 (57.8–76.5)	66.5 (58.0–79.0)	0.166
BMI	24.9 (22.0–29.3)	25.8 (22.2–29.9)	0.245
Mother ethnic origin			
White	234 (45%)	364 (40%)	0.129
Black	72 (14%)	163 (18%)	
Asian	96 (18%)	180 (20%)	
Mixed/other	129 (23%)	204 (22%)	
Nulliparous	282 (54%)	470 (52%)	0.193
Gestational age at booking	10 (9–12)	9 (8–11)	0.835
Blood pressure disorders	57 (11%)	105 (12%)	0.709
PTB < 34 weeks	174 (33%)	314 (34%)	0.627
Mode of PTB			
iPTB	265 (51%)	469 (51%)	0.740
sPTB	259 (49%)	442 (49%)	
Gestational age at delivery	35 (33–36)	35 (32–36)	0.724
Type of delivery			
Vaginal	348 (66%)	573 (63%)	0.377
Elective CS	38 (7%)	71 (8%)	
Emergency CS	137 (26%)	267 (29%)	
Neonatal weight (g)	2277 (1587–2692)	2200 (1460–2630)	0.434
Outcome			
Live birth	487 (93%)	827 (91%)	0.099
IUD	26 (5%)	65 (7%)	
NND	10 (2%)	11 (1%)	

*Note:* Numbers are presented as *n* (%), and median (IQR).

Abbreviations: %, percentage; BMI, body mass index; CS, caesareancesarean section; g, grams; iPTB, iatrogenic preterm births; IQR, interquartile; IUD, intrauterine death; kg, kilograms; *n*, numbers; NND, neonatal death; PTB, preterm births; sPTB, spontaneous preterm births.

**TABLE 2:** Interrupted time series analysis (ITSA) demonstrating the impact of the first‐trimester aspirin‐based screening and intervention program on the % of PTB, sPTB, and iPTB.

	Estimate (95% CI)	SE	*p*
% Total PTB			
Slope coefficient before the intervention	0.04 (−0.13 to 0.14)	0.06	0.95
Immediate effect of the intervention	−0.21 (−0.28 to 0.69)	0.24	0.39
Change in slopes between the pre‐ and post‐intervention	−0.01 (−0.15 to 0.13)	0.07	0.89
% sPTB			
Slope coefficient before the intervention	−0.06 (−0.13 to 0.01)	0.04	0.10
Immediate effect of the intervention	0.21 (−0.28 to 0.69)	0.24	0.39
Change in slopes between the pre‐ and post‐intervention	0.05 (−0.023 to 0.131)	0.037	0.16
% iPTB			
Slope coefficient before the intervention	0.06 (−0.05 to 0.18)	0.06	0.27
Immediate effect of the intervention	−0.44 (−1.24 to 0.35)	0.38	0.26
Change in slopes between the pre‐ and post‐intervention	−0.06 (−0.075 to −0.051)	0.06	0.30

Abbreviations: %, percentage; CI, confidence interval; iPTB, iatrogenic PTB; PTB, preterm birth; SE, standard error; sPTB, spontaneous preterm birth.

## 4 Discussion

### 4.1 Main Findings

The ITSA did not demonstrate a significant impact of targeted administration of aspirin based on the first‐trimester screening program on either iatrogenic or spontaneous preterm birth rates. The ITSA analysis indicated that there was a nonsignificant trend of reduction in PTB rates post‐screening implementation. There is an apparent stabilisation in the rate of iPTB and sPTB post‐intervention, whilst no trends are evident when only early PTBs are considered. These results are supported by a recent publication on a modelled cohort by Nicolaides et al. [11] that predicted no significant impact of aspirin on both iatrogenic and spontaneous PTB in a high‐risk population identified with the combined screening test for preterm preeclampsia.

### 4.2 Interpretation—First Trimester Combined Screening for Preeclampsia and PTB

Women identified as high risk for preterm preeclampsia in the first trimester are also at increased risk of developing other obstetric complications such as fetal growth restriction, stillbirth, and PTB [1, 7, 12]. Data from previous studies showed moderate detection for both iatrogenic [5] and sPTB [5, 13] with first‐trimester preeclampsia screening. However, it would be inappropriate to screen for pregnancies at increased risk of an adverse outcome, if it is not supported by a program of targeted intervention aimed to reduce its prevalence in the population.

### 4.3 Interpretation—Aspirin Prophylaxis and PTB

The PTB rate in the study population (4.5%) is consistent with that reported in most obstetric series and has remained stable over the years. The latter might reflect the fact that very few interventions have been shown to significantly reduce PTB rates. A Cochrane review including 35 212 women demonstrated that aspirin can significantly reduce the risk of PTB. The review included randomised controlled trials (RCTs) comparing the use of antiplatelet agents, such as aspirin, with a placebo in women at high risk of developing preterm preeclampsia. The findings of the Cochrane review indicated a 9% reduction in the relative risk of giving birth before 37 weeks among those who received antiplatelet therapy compared to those who received a placebo. However, the effect size is so small that treating 1000 high‐risk women with aspirin resulted in 159 cases of PTB compared to 175 in the placebo group—a reduction of 16 cases [9]. Biologically, there is an association between placental insufficiency and sPTB, providing a possible mechanism for aspirin playing a minor role in reducing the rate of sPTB. Furthermore, aspirin may lower the incidence of preterm preeclampsia, consequently decreasing the rate of iPTB [11]. However, the contributions of these specific reductions to the overall PTB rate are minimal, as shown in the findings of this study. Therefore, even with adherence to the aspirin regimen, the overall PTB rate is unlikely to be significantly impacted. This aligns with the findings of a recent study by Nicolaides et al. [11], which conducted a secondary analysis of the SPREE trial cohort and found that, in the modelled population, the impact of aspirin treatment on PTB in high‐risk pregnancy detected with the first trimester combined screening for preeclampsia is minimal or nonsignificant.

### 4.4 Strengths and Limitations

This study was conducted in a large tertiary centre unit, with high‐quality data collection. This setting allows for a detailed and reliable analysis of PTB rates and the impact of the first‐trimester screening program. Although this dataset is among the largest available, it still has proven to be insufficient to show any statistical power and, therefore, to draw definitive conclusions. Additionally, the study population was limited; no adjustments were made to account for demographic confounders, and the women included were followed up since the beginning of their pregnancies, which might have introduced intervention bias. These women could have received other specific interventions aimed at reducing PTBs, potentially confounding the results and making it challenging to isolate the effect of the screening program alone.

## 5 Conclusion

In summary, first‐trimester combined screening for preeclampsia identifies a group of women at higher risk of both sPTB and iPTB. Targeted intervention with aspirin prophylaxis and serial fetal well‐being assessment has been shown to reduce preterm preeclampsia and improve perinatal outcomes. However, there is no evidence of a significant effect on overall PTB rates in this population. Given the large population studied and the lack of a statistically significant impact, even if a minor effect exists, the number needed to treat before observing a meaningful reduction in PTB rates would be substantial. This suggests that even the widespread implementation of the first‐trimester screening aspirin‐based program alone may not be the most efficient strategy for PTB prevention. Instead, future efforts should focus on alternative or complementary interventions that may have a greater and more clinically significant impact on reducing PTB rates.

## Author Contributions

Conceptualisation: B.T. Methodology: B.T., A.P. Data collection and processing: M.M., A.M.‐V. Statistical analysis: M.M., A.M.‐V. Data interpretation: M.M., A.P., B.T. Manuscript draft: M.M., C.P., P.P. Manuscript review and editing: B.T., A.M.‐V., A.P., C.P., P.P., M.M.

## Funding

The authors have nothing to report.

## Ethics Statement

This retrospective study utilised routinely collected clinical data from an ongoing continuous audit and was determined not to require ethics approval or signed patient consent, in accordance with the HRA decision tool.

## Conflicts of Interest

The authors declare no conflicts of interest.

## Supporting information


**Appendix S1:** bjo70100‐sup‐0001‐Supinfo.docx.

## Data Availability

The data that support the findings of this study are available on request from the corresponding author. The data are not publicly available due to privacy or ethical restrictions.

## References

[1] D. L. Rolnik , R. J. Selvaratnam , D. Wertaschnigg , et al., “Routine First Trimester Combined Screening for Preterm Preeclampsia in Australia: A Multicenter Clinical Implementation Cohort Study,” International Journal of Gynaecology and Obstetrics 158, no. 3 (2022): 634–642. 34837224 10.1002/ijgo.14049

[2] D. L. Rolnik , D. Wright , L. C. Poon , et al., “Aspirin Versus Placebo in Pregnancies at High Risk for Preterm Preeclampsia,” New England Journal of Medicine 377, no. 7 (2017): 613–622. 28657417 10.1056/NEJMoa1704559

[3] L. C. Poon , D. L. Rolnik , M. Y. Tan , et al., “ASPRE Trial: Incidence of Preterm Pre‐Eclampsia in Patients Fulfilling ACOG and NICE Criteria According to Risk by FMF Algorithm,” Ultrasound in Obstetrics & Gynecology 51, no. 6 (2018): 738–742. 29380918 10.1002/uog.19019

[4] B. Liu , U. Nadeem , A. Frick , M. Alakaloko , A. Bhide , and B. Thilaganathan , “Reducing Health Inequality in Black, Asian and Other Minority Ethnic Pregnant Women: Impact of First Trimester Combined Screening for Placental Dysfunction on Perinatal Mortality,” BJOG 129, no. 10 (2022): 1750–1756. 35104381 10.1111/1471-0528.17109 PMC9544950

[5] V. Giorgione , O. Quintero Mendez , A. Pinas , W. Ansley , and B. Thilaganathan , “Routine First‐Trimester Pre‐Eclampsia Screening and Risk of Preterm Birth,” Ultrasound in Obstetrics & Gynecology 60, no. 2 (2022): 185–191. 35441764 10.1002/uog.24915 PMC9545360

[6] G. P. Guy , K. Leslie , D. Diaz Gomez , et al., “Effect of Routine First‐Trimester Combined Screening for Pre‐Eclampsia on Small‐For‐Gestational‐Age Birth: Secondary Interrupted Time Series Analysis,” Ultrasound in Obstetrics & Gynecology 59, no. 1 (2022): 55–60. 34319638 10.1002/uog.23741

[7] M. Minopoli , L. Noël , A. Meroni , M. Mascherpa , A. Frick , and B. Thilaganathan , “Adverse Pregnancy Outcomes in Women at Increased Risk of Preterm Pre‐Eclampsia on First‐Trimester Combined Screening,” BJOG: An International Journal of Obstetrics & Gynaecology 131 (2023): 81–87. 37271740 10.1111/1471-0528.17560

[8] G. P. Guy , K. Leslie , D. Diaz Gomez , et al., “Implementation of Routine First Trimester Combined Screening for Pre‐Eclampsia: A Clinical Effectiveness Study,” BJOG 128, no. 2 (2021): 149–156. 32613730 10.1111/1471-0528.16361

[9] L. Duley , S. Meher , K. E. Hunter , A. L. Seidler , L. M. Askie , and Cochrane Pregnancy and Childbirth Group , “Antiplatelet Agents for Preventing Pre‐Eclampsia and Its Complications,” Cochrane Database of Systematic Reviews 2019, no. 10 (2019): CD004659. 31684684 10.1002/14651858.CD004659.pub3 PMC6820858

[10] A. Linden , “Conducting Interrupted Time‐Series Analysis for Single‐ and Multiple‐Group Comparisons,” Stata Journal 15 (2015): 480–500.

[11] K. H. Nicolaides , A. Syngelaki , L. C. Poon , et al., “First‐Trimester Prediction of Preterm Pre‐Eclampsia and Prophylaxis by Aspirin: Effect on Spontaneous and Iatrogenic Preterm Birth,” BJOG 131, no. 4 (2024): 483–492. 37749709 10.1111/1471-0528.17673

[12] A. Boutin , P. Guerby , C. Gasse , S. Tapp , and E. Bujold , “Pregnancy Outcomes in Nulliparous Women With Positive First‐Trimester Preterm Preeclampsia Screening Test: The Great Obstetrical Syndromes Cohort Study,” American Journal of Obstetrics and Gynecology 224, no. 2 (2021): 204.e1–204.e7. 10.1016/j.ajog.2020.08.008 32777265

[13] P. I. Cavoretto , A. Farina , N. Salmeri , A. Syngelaki , M. Y. Tan , and K. H. Nicolaides , “First Trimester Risk of Preeclampsia and Rate of Spontaneous Birth in Patients Without Preeclampsia,” American Journal of Obstetrics and Gynecology 231 (2024): 452.e1–452.e7. 10.1016/j.ajog.2024.01.008 38244830

