# Supplementary material for: Effect of Targeted Aspirin Administration Based on First Trimester Combined Screening for Preeclampsia on Preterm Birth: An Interrupted Time Series Analysis
Source: BJOG. 2025 Nov 24;133(8):1547–52. doi: 10.1111/1471-0528.70100 (PMC13253998; doi:10.1111/1471-0528.70100)
Supplement: Supplementary file 1 — Appendix S1: bjo70100‐sup‐0001‐Supinfo.docx. [file BJO-133-1547-s001.docx]

**Table S1.** Interrupted time series analysis (ITSA) demonstrating the impact of the first-trimester aspirin-based screening and intervention program on the % of early PTB, sPTB, and iPTB < 34 weeks.

| **% Total PTB** | **Estimate (95% CI)** | **SE** | **p-value** |
| --- | --- | --- | --- |
| Slope coefficient before the intervention | -0.08 (-0.13 – 0.14) | 0.06 | 0.19 |
| Immediate effect of the intervention | 0.58 (-0.23 – 1.38) | 0.39 | 0.15 |
| Change in slopes between the pre- and post-intervention | 0.06 (-0.15 – 0.13) | 0.06 | 0.34 |
| **% sPTB** |  |  |  |
| Slope coefficient before the intervention | -0.07 (-0.12 – 0.01) | 0.03 | **0.02** |
| Immediate effect of the intervention | 0.26 (-0.10 – 0.62) | 0.18 | 0.15 |
| Change in slopes between the pre- and post-intervention | 0.05 (-0.01 – 0.11) | 0.03 | 0.07 |
| **% iPTB** |  |  |  |
| Slope coefficient before the intervention | -0.04 (-0.1 – 0.03) | 0.03 | 0.24 |
| Immediate effect of the intervention | 0.15 (-0.26 – 0.55) | 0.19 | 0.46 |
| Change in slopes between the pre- and post-intervention | 0.05 (-0.01 – 0.12) | 0.03 | 0.11 |

PTB, preterm birth; sPTB, spontaneous preterm birth; iPTB, iatrogenic PTB; %, percentage; CI confidence interval; SE, standard error

**Figure S1.** Time series of early (<34 weeks) PTB, iPTB, and sPTB rates presented in quarters of years (Q1-Q4) before and after the introduction of the first trimester combined screening program for preterm preeclampsia. The gray-shaded area represents the pre-intervention period (April 2016–July 2018). The dashed vertical line marks the introduction of the screening program. The dotted trend lines represent the trajectory of PTB, sPTB, and iPTB rates before and after the intervention. The change in intercept at the time of intervention reflects the immediate effect of the program, while the post-intervention trend indicates any long-term changes in PTB rates, as assessed through Interrupted Time Series Analysis (ITSA).
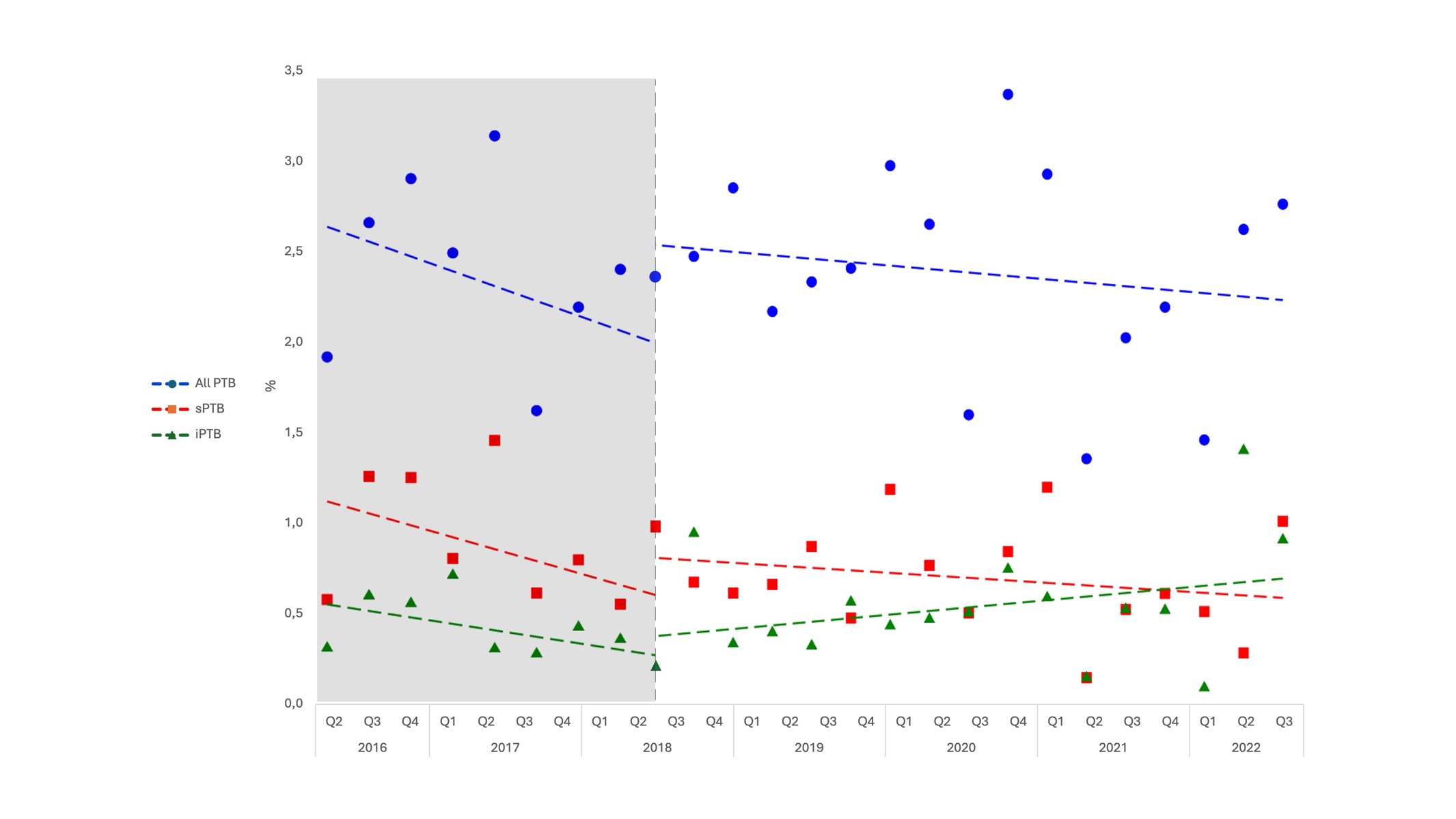


PTB, preterm birth; sPTB, spontaneous preterm birth; iPTB, iatrogenic preterm birth
